# Supra­molecular inter­actions in 2,6-di­amino-4-chloro­pyrimidin-1-ium 5-chloro­salicylate and bis­(2,6-di­amino-4-chloro­pyrimidin-1-ium) naphthalene-1,5-di­sulfonate

**DOI:** 10.1107/S2056989018001196

**Published:** 2018-01-26

**Authors:** Robert Swinton Darious, Packianathan Thomas Muthiah, Franc Perdih

**Affiliations:** a School of Chemistry, Bharathidasan University, Tiruchirappalli 620 024, Tamilnadu, India; b Faculty of Chemistry and Chemical Technology, University of Ljubljana, Večna, pot 113, PO Box 537, SI-1000 Ljubljana, Slovenia

**Keywords:** crystal structure, hydrogen bonding, supra­molecular architecture, halogen–halogen inter­action, quadruple array, homosynthon, heterosynthon

## Abstract

The crystals of two new salts, 2,6-di­amino-4-chloro­pyrimidin-1-ium 5-chloro­salicylate, C_4_H_6_ClN_4_^+^·C_7_H_4_ClO_3_^−^, (I), and bis­(2,6-di­amino-4-chloro­pyrimidin-1-ium) naphthalene-1,5-di-sulfonate, 2C_4_H_6_ClN_4_^+^·C_10_H_6_O_6_S_2_^2−^, (II), have been synthesized and characterized by single-crystal X-ray diffraction. In both compounds, the N atom of the pyrimidine group in between the amino substituents is protonated and the pyrimidinium cation forms a pair of N—H⋯O hydrogen bonds with the carboxyl­ate/sulfonate ion, leading to a robust *R*_2_^2^(8) motif (supra­molecular heterosynthon). In compound (I), a self-complementary base pairing involving the other pyrimidinium ring nitro­gen atom and one of the amino groups *via* a pair of N—H⋯N hydrogen bonds [*R*_2_^2^(8) homosynthon] is also present. In compound (II), the crystallographic inversion centre coincides with the inversion centre of the naphthalene-1,5-di­sulfonate ion and all the sulfonate O atoms are hydrogen-bond acceptors, generating fused-ring motifs and a quadruple *DDAA* array. A halogen-bond (Cl⋯Cl) inter­action is present in (I) with a distance and angle of 3.3505 (12) Å and 151.37 (10)°, respectively. In addition, a C—Cl⋯π inter­action and a π–π inter­action in (I) and a π–π inter­action in (II) further stabilize these crystal structures.

## Abstract 2

Two new salts – 2,6-di­amino-4-chloro­pyrimidin-1-ium 5-chloro­salicylate and bis­(2,6-di­amino-4-chloro­pyrimidin-1-ium) naphthalene-1,5-di­sulfonate – have been synthesized and characterized by single-crystal X-ray diffraction. The supra­molecular inter­actions such as hydrogen bonding, halogen bonding, C—Cl⋯π and π–π inter­actions are investigated for these crystal structures.

## Chemical context   

The study of supra­molecular inter­actions in the crystals of pyrimidinium salts continues to be an active field since the pyrimidine fragment is a component of nucleobases and many drug mol­ecules. The pyrimidine group offers two protonation sites (the two ring nitro­gens) and the site of protonation depends on the nature of the substituents. Tautomerism of the pyrimidinium cation has also been reported recently (Rajam *et al.*, 2017 ▸). The pyrimidinium–carboxyl­ate inter­action is also of fundamental importance in biology since it is involved in protein–nucleic acid inter­actions and drug-receptor recognition (Hunt *et al.*, 1980 ▸; Baker & Santi, 1965 ▸). The mol­ecules are often self-assembled by hydrogen bonding, halogen bonding, cation⋯π, anion⋯π and π–π stacking inter­actions. Among these inter­actions, halogen bonding is of particular current inter­est (Cavallo *et al.*, 2016 ▸). Various substituted pyrimidines and their inter­actions with different acids have been studied systematically in our laboratory. The variation in supra­molecular architectures resulting from the different substituents in the base and the acid is being investigated, and crystal structures of 2,6-di­amino-4-chloro­pyrimidinium salts with carboxyl­ate/sulfonate have been reported recently from our laboratory (Mohana *et al.*, 2017 ▸). The same pyrimidine derivative has been used to prepare the title compounds in order to further study the supra­molecular architectures and the role of the halogen bond.
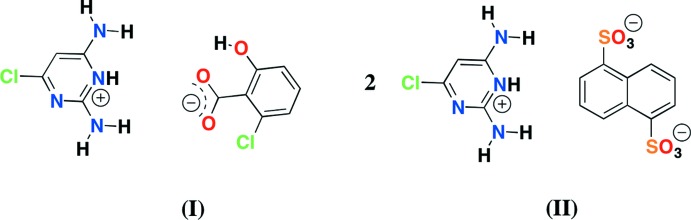


## Structural commentary   

**Figure 1:**
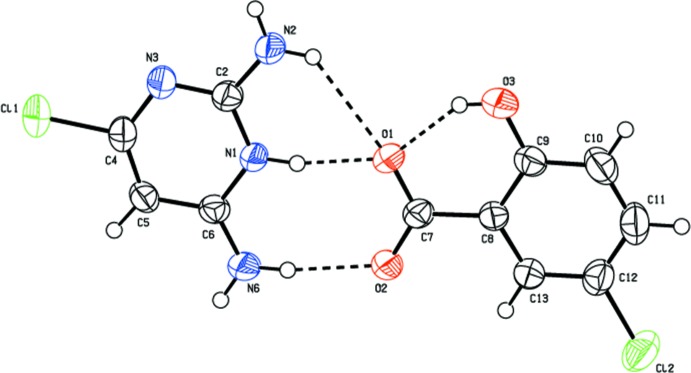
*ORTEP* view of compound (I) with the atom-numbering scheme. Displacement ellipsoids are drawn at 50% probability level. Dashed lines represent hydrogen bonds.

**Figure 2:**
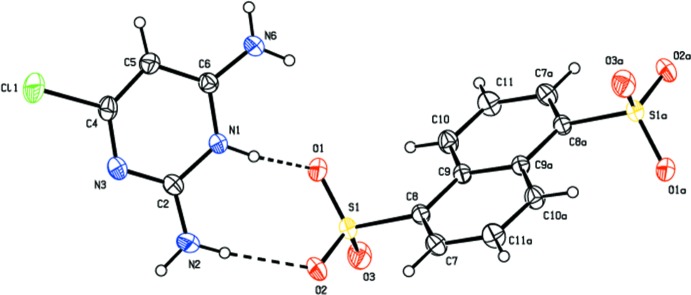
*ORTEP* view of compound (II), with the atom-numbering scheme. Displacement ellipsoids are drawn at 50% probability level. Dashed lines represent hydrogen bonds.

The salt of compound (I) crystallizes with one CDAPY (2,6-di­amino-4-chloro­pyrimidinium) cation and one CSA (5-chloro­salicylate) anion in the asymmetric unit (Fig. 1 ▸). The pyrimidinium cation is protonated at the N1 position (see Fig. 1 ▸ for atom numbering) and this is confirmed by an increase in the inter­nal bond angle. The C2—N3—C4 angle at the unprotonated N3 atom is 115.1 (2)°, while for the protonated N1 atom, the C2—N1—C6 angle is 121.8 (2)°. The ion-pair (CDAPY and CSA) is almost planar [dihedral angle = 4.22 (11)°]. The carboxyl­ate group of CSA is twisted slightly with respect to the remainder of the anion [dihedral angle= 3.9 (3)°]. The salt of compound (II) crystallizes with one CDAPY (2,6-di­amino-4-chloro­pyrimidinium) cation and half a mol­ecule of NSA (naphthalene-1,5-di­sulfonate) anion in the asymmetric unit (Fig. 2 ▸), the other half of NSA being generated by an inversion centre. A crystallographic inversion centre coinciding with the inversion centre of the NSA ion has also been reported earlier (Liu, 2012 ▸; Xu, 2012 ▸; Liu & Chen, 2012 ▸). The pyrimidinium cation is again protonated at the N1 position (see Fig. 2 ▸ for atom numbering) and this is confirmed by an increase in the inter­nal bond angle. The C2—N3—C4 angle at the unprotonated N3 atom is 115.40 (16)°, while the angle at the protonated N1 atom (C2—N1—C6) is 121.84 (16)°. All of the sulfonate oxygen atoms of the NSA anion are involved in hydrogen bonding. The S1—O1, S1—O2 and S1—O3 distances are similar [1.4550 (15), 1.4584 (15) and 1.4431 (16) Å respectively].

## Supra­molecular features   

**Figure 3:**
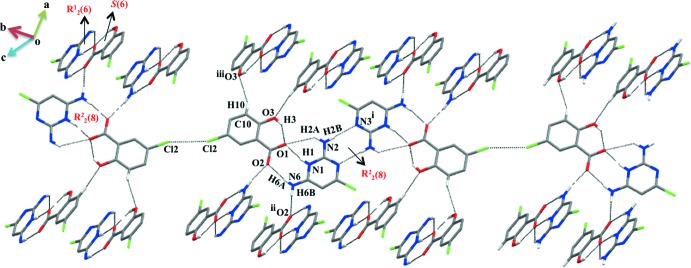
Supra­molecular layered structure extended as a chain *via* Cl⋯Cl inter­actions in (I).

**Figure 4:**
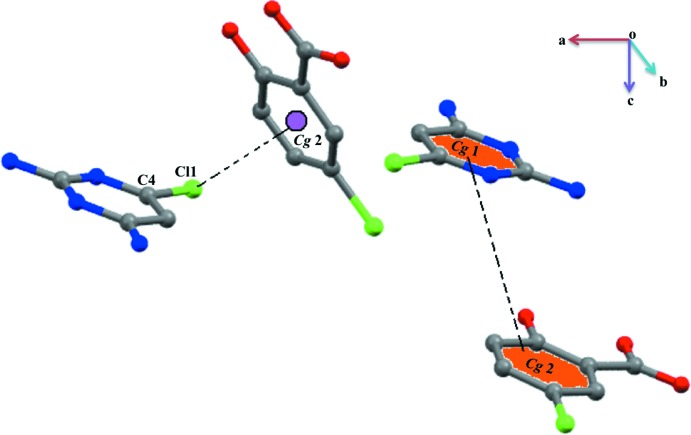
A weak C—Cl⋯π inter­action and π–π stacking inter­actions.

In salt (I), the protonated N1 atom and the amino hydrogen (N6) atom of CDAPY are hydrogen bonded *via* two N—H⋯O bonds (Table 1 ▸) forming a robust 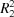
(8) ring motif (heterosynthon) involving the carboxyl­ate group. The typical intra­molecular hydrogen-bond *S*(6) motif (involving the carboxyl group and the phenolic –OH) observed in salicylates/salicylic acid is also present (Bernstein *et al.*, 1995 ▸; Prabakaran *et al.*, 2001 ▸; Panneerselvam *et al.*, 2002 ▸) (Fig. 1 ▸). The 2-amino hydrogen atom of CDAPY inter­acts with the carboxyl­ate oxygen O1 of CSA *via* an N—H⋯O hydrogen bond forming an 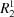
(6) ring motif. Thus, the O1 oxygen atom acts as a trifurcated acceptor. A similar set of three fused rings was observed in the crystal structure of 2,6-di­amino-4-chloro­pyrimidinium 2-carb­oxy-3-nitro­benzoate (Mohana *et al.*, 2017 ▸). However, in compound (I) the role of the 2-amino and 6-amino groups has been reversed. A self-complementary base pairing *via* a pair of N2—H⋯N3^i^ (homosynthon) hydrogen bonds forming an 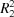
(8) ring motif is also been observed. This type of base pairing is also observed in the crystal structures of 2,6-di­amino-4-chloro­pyridinium 4-carb­oxy­butano­ate (Edison *et al.*, 2014 ▸), 2,6-di­amino-4-chloro­pyrimidine-benzoic acid (Thanigaimani *et al.*, 2012*a* ▸) and bis­(2,6-di­amino-4-chloro­pyrimidin-1-ium) fumarate (Thanigaimani *et al.*, 2012*b* ▸). The 2,6-di­amino-4-chloro­pyrimidinium 5-chloro­salicylate units are linked *via* a Cl⋯Cl inter­action (a type I inter­action; Cavallo *et al.*, 2016 ▸) with a distance and angle of 3.3505 (12) Å and 151.37 (10)°, respectively (Durka *et al.*, 2015 ▸) (Fig. 3 ▸). Furthermore, a weak C—H⋯O^iii^ hydrogen-bonding inter­action is present in this crystal structure. In addition, a weak stacking inter­action with *Cg*1⋯*Cg*2 [3.6624 (14) Å; symmetry code: *x*, −1 + *y*, *z*; *Cg*1 and *Cg*2 are the centroids of the N1/C2/N3/C4/C5/C6 and C8–C13 rings, respectively] and C—Cl⋯π inter­actions [3.4469 (13) Å with an angle of 152.24 (9)°; symmetry code: −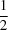 + *x*, 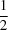 − *y*, −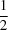 + *z*] (Muthukumaran *et al.*, 2011 ▸) further stabilize this crystal structure (Fig. 4 ▸).

**Figure 5:**
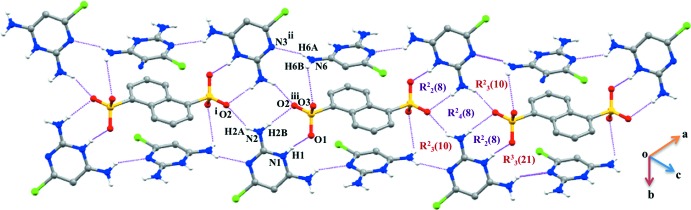
Formation of a quadruple *DDAA* array in (II) *via* N—H⋯O hydrogen bonds.

**Figure 6:**
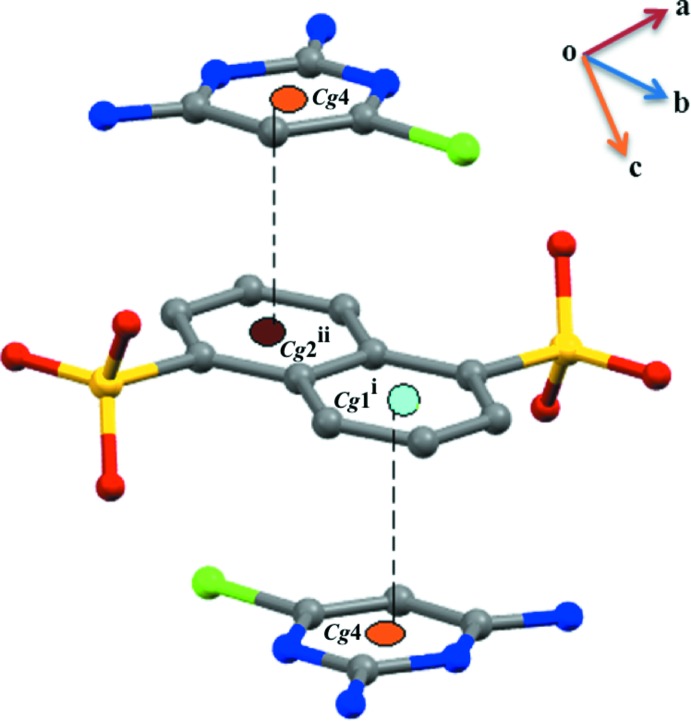
A view of the π–π stacking inter­actions between the pyrimidinium cation and the anion.

In salt (II), the sulfonate group mimics the role of the carboxyl­ate oxygen atoms in generating an 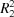
(8) motif (heterosynthon) involving the amino­pyrimidinium cation (CDAPY) (Bernstein *et al.*, 1995 ▸; Balasubramani *et al.*, 2007 ▸). All units of the CDAPY and NSA ions are hydrogen bonded (Table 2 ▸) to generate a quadruple DDAA array with fused ring motifs 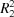
(8), 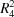
(8) and 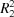
(8) (Fig. 5 ▸). This type of array has also been reported earlier (Robert *et al.*, 2001 ▸; Umadevi *et al.*, 2002 ▸; Raj *et al.*, 2003 ▸; Subashini *et al.*, 2007 ▸; Thanigaimani *et al.*, 2007 ▸; Liu & Chen, 2012 ▸). In addition, the NSA anions also generate 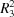
(10) and 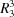
(21) ring motifs *via* N—H⋯O bonds. Weak π–π stacking inter­actions [*Cg*1⋯*Cg*4 = 3.4781 (11) Å; symmetry code: 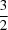 − *x*, −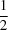 + *y*, 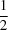 − *z* and *Cg*4⋯*Cg*2 =3.4781 (11) Å; symmetry code: 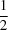 + *x*, 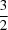 − *y*, 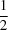 + *z*; *Cg*1, *Cg*2 and *Cg*4 are the centroids of the C7/C8/C9/C9′/C10′/C11′, C9/C10/C11/C7′/C8′/C9′ and N1/C2/N3/C4/C5/C6 rings, respectively] is also present (Fig. 6 ▸).

## Database survey   

Various salts of 5-chloro­salicylate have been reported: 2-methyl­quinolinium 5-chloro-2-hy­droxy­benzoate (Zhang *et al.*, 2014 ▸), 4-amino-5-chloro-2,6-di­methyl­pyrimidinium 5-chloro-2-hy­droxy­benzoate (Rajam *et al.*, 2017 ▸) and 2-amino-4,6-di­methyl­pyrimidinium 5-chloro­salicylate (Ebenezer & Mu­thiah, 2012 ▸). Similarly, various salts of half a mol­ecule of naphthalene-1,5-di­sulfonate have been reported: bis­(2-tri­fluoro­methyl-1*H*-benzimidazole-3-ium) naphthalene-1,5-di­sulfonate (Liu, 2012 ▸), bis­(3-methyl­anilinium) naphthalene-1,5-di­sulfonate (Liu & Chen, 2012 ▸) and bis­(2-methyl­piperidinium) naphthalene-1,5-di­sulfonate (Xu, 2012 ▸).

## Synthesis and crystallization   

Compounds (I) and (II) were synthesized by mixing hot ethano­lic solutions (1:1) of 2,6-di­amino-4-chloro­pyrimidine (36 mg) with 5-chloro­salicylic acid (43 mg) (I)/naphthalene-1,5-di­sulfonic acid (72 mg) (II). These mixtures were warmed to 333 K for 25 min. Colourless crystals separated out from the mother liquor at room temperature after a week.

## Refinement   

Crystal data, data collection and structure refinement details are summarized in Table 3 ▸. All H atoms were initially located readily in difference-Fourier maps and were treated as riding atoms with C—H = 0.93 Å (aromatic), N—H = 0.86 Å and O—H = 0.82 Å with *U*_iso_(H) = *kU*_eq_(C,N,O), where *k* = 1.5 for hy­droxy and 1.2 for all other H atoms.

## Supplementary Material

Crystal structure: contains datablock(s) I, II. DOI: 10.1107/S2056989018001196/zl2723sup1.cif

Structure factors: contains datablock(s) I. DOI: 10.1107/S2056989018001196/zl2723Isup2.hkl

Structure factors: contains datablock(s) II. DOI: 10.1107/S2056989018001196/zl2723IIsup3.hkl

Supporting information file. DOI: 10.1107/S2056989018001196/zl2723Isup4.cml

CCDC references: 1817972, 1817971

Additional supporting information:  crystallographic information; 3D view; checkCIF report

## Figures and Tables

**Table 1:** Hydrogen-bond geometry (Å, °) for (I)

*D*—H⋯*A*	*D*—H	H⋯*A*	*D*⋯*A*	*D*—H⋯*A*
N1—H1⋯O1	0.86	1.82	2.664 (3)	168
N2—H2*A*⋯O1	0.86	2.56	3.223 (3)	135
N2—H2*B*⋯N3^i^	0.86	2.13	2.970 (3)	165
O3—H3⋯O1	0.82	1.83	2.557 (3)	146
N6—H6*A*⋯O2	0.86	1.97	2.824 (3)	172
N6—H6*B*⋯O2^ii^	0.86	1.96	2.819 (3)	172
C10—H10⋯O3^iii^	0.93	2.51	3.358 (4)	151

Symmetry codes: (i) 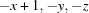; (ii) 
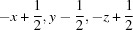
; (iii) 
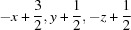
.

**Table 2:** Hydrogen-bond geometry (Å, °) for (II)

*D*—H⋯*A*	*D*—H	H⋯*A*	*D*⋯*A*	*D*—H⋯*A*
N1—H1⋯O1	0.86	1.92	2.708 (2)	152
N2—H2*A*⋯O2^i^	0.86	2.08	2.868 (3)	152
N2—H2*B*⋯O2	0.86	2.10	2.953 (2)	174
N6—H6*A*⋯N3^ii^	0.86	2.25	2.943 (2)	138
N6—H6*B*⋯O3^iii^	0.86	2.01	2.808 (2)	154

Symmetry codes: (i) 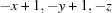; (ii) 
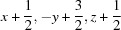
; (iii) 
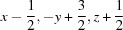
.

**Table 3:** Experimental details

	(I)	(II)
Crystal data		
Chemical formula	C_4_H_6_ClN_4_^+^·C_7_H_4_ClO_3_^−^	2C_4_H_6_ClN_4_^+^·C_10_H_6_O_6_S_2_^2−^
*M*_r_	317.13	577.42
Crystal system, space group	Monoclinic, *P*2_1_/*n*	Monoclinic, *P*2_1_/*n*
Temperature (K)	293	293
*a*, *b*, *c* (Å)	13.9203 (14), 7.0285 (6), 15.4294 (14)	9.1696 (4), 13.0848 (7), 9.9663 (5)
β (°)	114.544 (12)	90.526 (5)
*V* (Å^3^)	1373.2 (3)	1195.73 (10)
*Z*	4	2
Radiation type	Mo *K*α	Mo *K*α
μ (mm^−1^)	0.49	0.50
Crystal size (mm)	0.40 × 0.10 × 0.03	0.40 × 0.40 × 0.06

Data collection		
Diffractometer	Agilent SuperNova Dual Source diffractometer with an Atlas detector	Agilent SuperNova Dual Source diffractometer with an Atlas detector
Absorption correction	Multi-scan (*CrysAlis PRO*); Agilent, 2013 ▸)	Multi-scan (*CrysAlis PRO*; Agilent, 2013 ▸)
*T*_min_, *T*_max_	0.644, 1.000	0.527, 1.000
No. of measured, independent and observed [*I* > 2σ(*I*)] reflections	7906, 3144, 2137	10382, 2735, 2274
*R*_int_	0.027	0.028
(sin θ/λ)_max_ (Å^−1^)	0.649	0.649

Refinement		
*R*[*F* ^2^ > 2σ(*F* ^2^)], *wR*(*F* ^2^), *S*	0.048, 0.128, 1.04	0.038, 0.102, 1.05
No. of reflections	3144	2735
No. of parameters	182	163
H-atom treatment	H-atom parameters constrained	H-atom parameters constrained
Δρ_max_, Δρ_min_ (e Å^−3^)	0.29, −0.40	0.49, −0.59

Computer programs: *CrysAlis PRO* (Agilent, 2013 ▸), *SHELXT2014* (Sheldrick, 2015*a* ▸), *SHELXL2014* (Sheldrick, 2015*b* ▸), *PLATON* (Spek, 2009 ▸) and *Mercury* (Macrae *et al.*, 2008 ▸).

## supplementary crystallographic information

### 2,6-Diamino-4-chloropyrimidin-1-ium 2-chloro-6-hydroxybenzoate (I) Crystal data

**Table:**

C_4_H_6_ClN_4_^+^·C_7_H_4_ClO_3_^−^	*F*(000) = 648
*M**_r_* = 317.13	*D*_x_ = 1.534 Mg m^−^^3^
Monoclinic, *P*2_1_/*n*	Mo *K*α radiation, λ = 0.71073 Å
*a* = 13.9203 (14) Å	Cell parameters from 1734 reflections
*b* = 7.0285 (6) Å	θ = 3.9–27.5°
*c* = 15.4294 (14) Å	µ = 0.49 mm^−^^1^
β = 114.544 (12)°	*T* = 293 K
*V* = 1373.2 (3) Å^3^	Needle, colorless
*Z* = 4	0.40 × 0.10 × 0.03 mm

### 2,6-Diamino-4-chloropyrimidin-1-ium 2-chloro-6-hydroxybenzoate (I) Data collection

**Table:**

Agilent SuperNova Dual Source diffractometer with an Atlas detector	3144 independent reflections
Radiation source: SuperNova (Mo) X-ray Source	2137 reflections with *I* > 2σ(*I*)
Mirror monochromator	*R*_int_ = 0.027
Detector resolution: 10.4933 pixels mm^-1^	θ_max_ = 27.5°, θ_min_ = 2.9°
ω scans	*h* = −18→17
Absorption correction: multi-scan (CrysAlis PRO); Agilent, 2013)	*k* = −7→9
*T*_min_ = 0.644, *T*_max_ = 1.000	*l* = −20→19
7906 measured reflections	

### 2,6-Diamino-4-chloropyrimidin-1-ium 2-chloro-6-hydroxybenzoate (I) Refinement

**Table:**

Refinement on *F*^2^	0 restraints
Least-squares matrix: full	Hydrogen site location: inferred from neighbouring sites
*R*[*F*^2^ > 2σ(*F*^2^)] = 0.048	H-atom parameters constrained
*wR*(*F*^2^) = 0.128	*w* = 1/[σ^2^(*F*_o_^2^) + (0.0482*P*)^2^ + 0.5033*P*] where *P* = (*F*_o_^2^ + 2*F*_c_^2^)/3
*S* = 1.04	(Δ/σ)_max_ < 0.001
3144 reflections	Δρ_max_ = 0.29 e Å^−^^3^
182 parameters	Δρ_min_ = −0.40 e Å^−^^3^

### 2,6-Diamino-4-chloropyrimidin-1-ium 2-chloro-6-hydroxybenzoate (I) Special details

**Table:**

Geometry. All esds (except the esd in the dihedral angle between two l.s. planes) are estimated using the full covariance matrix. The cell esds are taken into account individually in the estimation of esds in distances, angles and torsion angles; correlations between esds in cell parameters are only used when they are defined by crystal symmetry. An approximate (isotropic) treatment of cell esds is used for estimating esds involving l.s. planes.

### 2,6-Diamino-4-chloropyrimidin-1-ium 2-chloro-6-hydroxybenzoate (I) Fractional atomic coordinates and isotropic or equivalent isotropic displacement parameters (Å^2^)

**Table:**

	*x*	*y*	*z*	*U*_iso_*/*U*_eq_	
Cl1	0.25461 (6)	−0.27636 (11)	−0.00641 (5)	0.0674 (2)	
N1	0.38557 (14)	0.2707 (3)	0.12207 (13)	0.0386 (4)	
H1	0.4128	0.3792	0.1450	0.046*	
N2	0.50448 (17)	0.2485 (3)	0.05504 (16)	0.0566 (6)	
H2B	0.5313	0.1895	0.0215	0.068*	
H2A	0.5293	0.3573	0.0796	0.068*	
N3	0.38700 (15)	0.0032 (3)	0.03064 (13)	0.0442 (5)	
N6	0.27205 (16)	0.3072 (3)	0.19347 (15)	0.0503 (5)	
H6A	0.3023	0.4141	0.2157	0.060*	
H6B	0.2207	0.2687	0.2063	0.060*	
C2	0.42485 (18)	0.1715 (3)	0.06889 (16)	0.0404 (5)	
C4	0.30522 (17)	−0.0606 (3)	0.04713 (16)	0.0414 (5)	
C5	0.26096 (16)	0.0273 (3)	0.09993 (15)	0.0398 (5)	
H5	0.2048	−0.0266	0.1089	0.048*	
C6	0.30401 (16)	0.2033 (3)	0.14025 (15)	0.0368 (5)	
Cl2	0.50995 (6)	1.29096 (12)	0.45221 (5)	0.0745 (3)	
O1	0.49261 (13)	0.5917 (2)	0.18881 (13)	0.0534 (5)	
O2	0.38734 (12)	0.6467 (2)	0.26174 (12)	0.0501 (4)	
O3	0.62982 (14)	0.8382 (3)	0.19368 (15)	0.0617 (5)	
H3	0.5981	0.7365	0.1805	0.093*	
C7	0.46259 (17)	0.6930 (3)	0.24184 (16)	0.0393 (5)	
C8	0.51962 (15)	0.8751 (3)	0.27862 (15)	0.0362 (5)	
C9	0.60003 (17)	0.9377 (4)	0.25339 (17)	0.0437 (6)	
C10	0.65092 (19)	1.1105 (4)	0.28915 (19)	0.0563 (7)	
H10	0.7038	1.1527	0.2718	0.068*	
C11	0.6235 (2)	1.2181 (4)	0.34943 (19)	0.0580 (7)	
H11	0.6576	1.3330	0.3729	0.070*	
C12	0.54500 (19)	1.1548 (4)	0.37507 (17)	0.0488 (6)	
C13	0.49410 (17)	0.9865 (3)	0.34066 (16)	0.0412 (5)	
H13	0.4417	0.9458	0.3590	0.049*	

### 2,6-Diamino-4-chloropyrimidin-1-ium 2-chloro-6-hydroxybenzoate (I) Atomic displacement parameters (Å^2^)

**Table:**

	*U*^11^	*U*^22^	*U*^33^	*U*^12^	*U*^13^	*U*^23^
Cl1	0.0648 (4)	0.0561 (5)	0.0783 (5)	−0.0240 (3)	0.0267 (4)	−0.0292 (4)
N1	0.0432 (10)	0.0303 (10)	0.0503 (10)	−0.0045 (8)	0.0274 (9)	−0.0059 (9)
N2	0.0670 (13)	0.0480 (13)	0.0795 (15)	−0.0198 (11)	0.0551 (12)	−0.0247 (12)
N3	0.0479 (11)	0.0401 (11)	0.0490 (11)	−0.0089 (9)	0.0245 (9)	−0.0100 (10)
N6	0.0527 (11)	0.0418 (12)	0.0747 (14)	−0.0065 (10)	0.0448 (11)	−0.0087 (11)
C2	0.0460 (12)	0.0383 (13)	0.0442 (12)	−0.0032 (11)	0.0258 (10)	−0.0027 (11)
C4	0.0403 (12)	0.0346 (13)	0.0421 (11)	−0.0045 (10)	0.0099 (10)	−0.0031 (11)
C5	0.0337 (11)	0.0385 (13)	0.0477 (12)	−0.0050 (10)	0.0176 (10)	0.0007 (11)
C6	0.0354 (11)	0.0341 (12)	0.0433 (11)	0.0029 (9)	0.0187 (10)	0.0030 (10)
Cl2	0.0751 (5)	0.0685 (5)	0.0735 (5)	0.0006 (4)	0.0245 (4)	−0.0334 (4)
O1	0.0566 (10)	0.0395 (10)	0.0798 (12)	−0.0057 (8)	0.0439 (9)	−0.0171 (9)
O2	0.0519 (10)	0.0380 (9)	0.0762 (11)	−0.0081 (8)	0.0425 (9)	−0.0081 (9)
O3	0.0555 (11)	0.0592 (13)	0.0892 (13)	−0.0074 (9)	0.0487 (10)	−0.0126 (11)
C7	0.0401 (12)	0.0314 (12)	0.0494 (12)	0.0037 (10)	0.0216 (10)	0.0012 (10)
C8	0.0315 (10)	0.0326 (12)	0.0427 (11)	0.0022 (9)	0.0136 (9)	0.0001 (10)
C9	0.0351 (11)	0.0427 (14)	0.0539 (13)	0.0011 (10)	0.0192 (10)	0.0002 (12)
C10	0.0436 (13)	0.0566 (17)	0.0695 (16)	−0.0126 (13)	0.0242 (13)	−0.0008 (15)
C11	0.0517 (15)	0.0456 (15)	0.0638 (16)	−0.0121 (13)	0.0112 (13)	−0.0105 (14)
C12	0.0451 (13)	0.0436 (14)	0.0485 (13)	0.0009 (11)	0.0102 (11)	−0.0088 (12)
C13	0.0356 (11)	0.0399 (13)	0.0466 (12)	0.0005 (10)	0.0156 (10)	−0.0032 (11)

### 2,6-Diamino-4-chloropyrimidin-1-ium 2-chloro-6-hydroxybenzoate (I) Geometric parameters (Å, º)

**Table:**

Cl1—C4	1.731 (2)	Cl2—C12	1.747 (3)
N1—C2	1.353 (3)	O1—C7	1.279 (3)
N1—C6	1.362 (3)	O2—C7	1.251 (3)
N1—H1	0.8600	O3—C9	1.352 (3)
N2—C2	1.328 (3)	O3—H3	0.8200
N2—H2B	0.8600	C7—C8	1.488 (3)
N2—H2A	0.8600	C8—C13	1.392 (3)
N3—C2	1.329 (3)	C8—C9	1.400 (3)
N3—C4	1.342 (3)	C9—C10	1.399 (4)
N6—C6	1.307 (3)	C10—C11	1.370 (4)
N6—H6A	0.8600	C10—H10	0.9300
N6—H6B	0.8600	C11—C12	1.382 (4)
C4—C5	1.357 (3)	C11—H11	0.9300
C5—C6	1.402 (3)	C12—C13	1.368 (3)
C5—H5	0.9300	C13—H13	0.9300
			
C2—N1—C6	121.80 (19)	C9—O3—H3	109.5
C2—N1—H1	119.1	O2—C7—O1	122.8 (2)
C6—N1—H1	119.1	O2—C7—C8	119.9 (2)
C2—N2—H2B	120.0	O1—C7—C8	117.29 (19)
C2—N2—H2A	120.0	C13—C8—C9	118.4 (2)
H2B—N2—H2A	120.0	C13—C8—C7	119.85 (19)
C2—N3—C4	115.1 (2)	C9—C8—C7	121.7 (2)
C6—N6—H6A	120.0	O3—C9—C10	118.0 (2)
C6—N6—H6B	120.0	O3—C9—C8	122.3 (2)
H6A—N6—H6B	120.0	C10—C9—C8	119.7 (2)
N2—C2—N3	119.6 (2)	C11—C10—C9	120.6 (2)
N2—C2—N1	117.5 (2)	C11—C10—H10	119.7
N3—C2—N1	122.8 (2)	C9—C10—H10	119.7
N3—C4—C5	126.4 (2)	C10—C11—C12	119.5 (2)
N3—C4—Cl1	114.28 (18)	C10—C11—H11	120.2
C5—C4—Cl1	119.28 (18)	C12—C11—H11	120.2
C4—C5—C6	116.8 (2)	C13—C12—C11	120.7 (2)
C4—C5—H5	121.6	C13—C12—Cl2	119.5 (2)
C6—C5—H5	121.6	C11—C12—Cl2	119.8 (2)
N6—C6—N1	117.7 (2)	C12—C13—C8	121.0 (2)
N6—C6—C5	125.3 (2)	C12—C13—H13	119.5
N1—C6—C5	117.0 (2)	C8—C13—H13	119.5
			
C4—N3—C2—N2	−178.7 (2)	O1—C7—C8—C9	2.6 (3)
C4—N3—C2—N1	1.2 (3)	C13—C8—C9—O3	179.9 (2)
C6—N1—C2—N2	−180.0 (2)	C7—C8—C9—O3	0.7 (3)
C6—N1—C2—N3	0.2 (3)	C13—C8—C9—C10	−1.2 (3)
C2—N3—C4—C5	−1.7 (3)	C7—C8—C9—C10	179.5 (2)
C2—N3—C4—Cl1	178.08 (16)	O3—C9—C10—C11	179.5 (2)
N3—C4—C5—C6	0.9 (3)	C8—C9—C10—C11	0.7 (4)
Cl1—C4—C5—C6	−178.93 (16)	C9—C10—C11—C12	0.1 (4)
C2—N1—C6—N6	179.0 (2)	C10—C11—C12—C13	−0.2 (4)
C2—N1—C6—C5	−1.1 (3)	C10—C11—C12—Cl2	179.8 (2)
C4—C5—C6—N6	−179.5 (2)	C11—C12—C13—C8	−0.3 (4)
C4—C5—C6—N1	0.6 (3)	Cl2—C12—C13—C8	179.61 (17)
O2—C7—C8—C13	4.7 (3)	C9—C8—C13—C12	1.1 (3)
O1—C7—C8—C13	−176.68 (19)	C7—C8—C13—C12	−179.7 (2)
O2—C7—C8—C9	−176.1 (2)		

### 2,6-Diamino-4-chloropyrimidin-1-ium 2-chloro-6-hydroxybenzoate (I) Hydrogen-bond geometry (Å, º)

**Table:**

*D*—H···*A*	*D*—H	H···*A*	*D*···*A*	*D*—H···*A*
N1—H1···O1	0.86	1.82	2.664 (3)	168
N2—H2*A*···O1	0.86	2.56	3.223 (3)	135
N2—H2*B*···N3^i^	0.86	2.13	2.970 (3)	165
O3—H3···O1	0.82	1.83	2.557 (3)	146
N6—H6*A*···O2	0.86	1.97	2.824 (3)	172
N6—H6*B*···O2^ii^	0.86	1.96	2.819 (3)	172
C10—H10···O3^iii^	0.93	2.51	3.358 (4)	151

Symmetry codes: (i) −*x*+1, −*y*, −*z*; (ii) −*x*+1/2, *y*−1/2, −*z*+1/2; (iii) −*x*+3/2, *y*+1/2, −*z*+1/2.

### Bis(2,6-diamino-4-chloropyrimidin-1-ium) naphthalene-1,5-disulfonate (II) Crystal data

**Table:**

2C_4_H_6_ClN_4_^+^·C_10_H_6_O_6_S_2_^2^^−^	*F*(000) = 592
*M**_r_* = 577.42	*D*_x_ = 1.604 Mg m^−^^3^
Monoclinic, *P*2_1_/*n*	Mo *K*α radiation, λ = 0.71073 Å
*a* = 9.1696 (4) Å	Cell parameters from 3749 reflections
*b* = 13.0848 (7) Å	θ = 3.7–30.1°
*c* = 9.9663 (5) Å	µ = 0.50 mm^−^^1^
β = 90.526 (5)°	*T* = 293 K
*V* = 1195.73 (10) Å^3^	Prism, colorless
*Z* = 2	0.40 × 0.40 × 0.06 mm

### Bis(2,6-diamino-4-chloropyrimidin-1-ium) naphthalene-1,5-disulfonate (II) Data collection

**Table:**

Agilent SuperNova Dual Source diffractometer with an Atlas detector	2735 independent reflections
Radiation source: SuperNova (Mo) X-ray Source	2274 reflections with *I* > 2σ(*I*)
Mirror monochromator	*R*_int_ = 0.028
Detector resolution: 10.4933 pixels mm^-1^	θ_max_ = 27.5°, θ_min_ = 3.0°
ω scans	*h* = −8→11
Absorption correction: multi-scan (CrysAlis PRO; Agilent, 2013)	*k* = −16→15
*T*_min_ = 0.527, *T*_max_ = 1.000	*l* = −12→12
10382 measured reflections	

### Bis(2,6-diamino-4-chloropyrimidin-1-ium) naphthalene-1,5-disulfonate (II) Refinement

**Table:**

Refinement on *F*^2^	0 restraints
Least-squares matrix: full	Hydrogen site location: inferred from neighbouring sites
*R*[*F*^2^ > 2σ(*F*^2^)] = 0.038	H-atom parameters constrained
*wR*(*F*^2^) = 0.102	*w* = 1/[σ^2^(*F*_o_^2^) + (0.0444*P*)^2^ + 0.5881*P*] where *P* = (*F*_o_^2^ + 2*F*_c_^2^)/3
*S* = 1.05	(Δ/σ)_max_ < 0.001
2735 reflections	Δρ_max_ = 0.49 e Å^−^^3^
163 parameters	Δρ_min_ = −0.59 e Å^−^^3^

### Bis(2,6-diamino-4-chloropyrimidin-1-ium) naphthalene-1,5-disulfonate (II) Special details

**Table:**

Geometry. All esds (except the esd in the dihedral angle between two l.s. planes) are estimated using the full covariance matrix. The cell esds are taken into account individually in the estimation of esds in distances, angles and torsion angles; correlations between esds in cell parameters are only used when they are defined by crystal symmetry. An approximate (isotropic) treatment of cell esds is used for estimating esds involving l.s. planes.

### Bis(2,6-diamino-4-chloropyrimidin-1-ium) naphthalene-1,5-disulfonate (II) Fractional atomic coordinates and isotropic or equivalent isotropic displacement parameters (Å^2^)

**Table:**

	*x*	*y*	*z*	*U*_iso_*/*U*_eq_	
Cl1	0.10474 (6)	0.91005 (5)	0.13497 (8)	0.0693 (2)	
N1	0.47951 (17)	0.72194 (12)	0.24106 (15)	0.0379 (4)	
H1	0.5541	0.6839	0.2572	0.045*	
N2	0.4259 (2)	0.61789 (16)	0.0635 (2)	0.0675 (7)	
H2A	0.3734	0.6006	−0.0047	0.081*	
H2B	0.5013	0.5823	0.0853	0.081*	
N3	0.27559 (18)	0.75496 (14)	0.10314 (17)	0.0437 (4)	
N6	0.54208 (19)	0.81915 (14)	0.42350 (17)	0.0444 (4)	
H6A	0.6148	0.7789	0.4376	0.053*	
H6B	0.5275	0.8701	0.4761	0.053*	
C2	0.3908 (2)	0.69909 (16)	0.1347 (2)	0.0420 (5)	
C4	0.2528 (2)	0.83590 (15)	0.1817 (2)	0.0393 (4)	
C5	0.3328 (2)	0.86453 (15)	0.2911 (2)	0.0377 (4)	
H5	0.3089	0.9215	0.3422	0.045*	
C6	0.45350 (19)	0.80277 (14)	0.32183 (18)	0.0333 (4)	
S1	0.80386 (5)	0.55638 (4)	0.21480 (4)	0.03723 (15)	
O1	0.75993 (16)	0.65802 (11)	0.25770 (15)	0.0491 (4)	
O2	0.68017 (17)	0.49952 (12)	0.15999 (15)	0.0517 (4)	
O3	0.92854 (18)	0.55687 (13)	0.12748 (14)	0.0541 (4)	
C7	0.7936 (2)	0.40041 (15)	0.39206 (19)	0.0379 (4)	
H7	0.7184	0.3761	0.3375	0.045*	
C8	0.86088 (18)	0.49007 (14)	0.36151 (17)	0.0307 (4)	
C9	0.97829 (18)	0.52894 (13)	0.44251 (17)	0.0293 (4)	
C10	1.0523 (2)	0.62150 (15)	0.41375 (19)	0.0385 (4)	
H10	1.0250	0.6595	0.3388	0.046*	
C11	1.1626 (2)	0.65540 (16)	0.4944 (2)	0.0427 (5)	
H11	1.2097	0.7163	0.4739	0.051*	

### Bis(2,6-diamino-4-chloropyrimidin-1-ium) naphthalene-1,5-disulfonate (II) Atomic displacement parameters (Å^2^)

**Table:**

	*U*^11^	*U*^22^	*U*^33^	*U*^12^	*U*^13^	*U*^23^
Cl1	0.0446 (3)	0.0740 (4)	0.0888 (5)	0.0223 (3)	−0.0221 (3)	−0.0052 (4)
N1	0.0362 (8)	0.0366 (8)	0.0405 (8)	0.0056 (7)	−0.0128 (7)	−0.0061 (7)
N2	0.0709 (13)	0.0633 (13)	0.0676 (13)	0.0230 (11)	−0.0364 (11)	−0.0346 (11)
N3	0.0371 (9)	0.0485 (10)	0.0454 (9)	0.0019 (7)	−0.0144 (7)	−0.0041 (8)
N6	0.0459 (10)	0.0451 (9)	0.0420 (9)	0.0073 (8)	−0.0150 (8)	−0.0107 (7)
C2	0.0415 (11)	0.0420 (11)	0.0424 (10)	0.0010 (8)	−0.0122 (9)	−0.0066 (8)
C4	0.0275 (9)	0.0429 (11)	0.0474 (11)	0.0012 (8)	−0.0047 (8)	0.0052 (9)
C5	0.0347 (9)	0.0367 (10)	0.0416 (10)	0.0032 (8)	−0.0022 (8)	−0.0028 (8)
C6	0.0327 (9)	0.0338 (9)	0.0333 (9)	−0.0023 (7)	−0.0023 (7)	0.0000 (7)
S1	0.0411 (3)	0.0401 (3)	0.0303 (2)	0.0063 (2)	−0.00909 (19)	0.00095 (18)
O1	0.0499 (8)	0.0425 (8)	0.0545 (9)	0.0128 (7)	−0.0185 (7)	−0.0033 (7)
O2	0.0563 (9)	0.0532 (9)	0.0451 (8)	0.0030 (7)	−0.0252 (7)	−0.0052 (7)
O3	0.0625 (10)	0.0643 (10)	0.0356 (8)	0.0090 (8)	0.0075 (7)	0.0121 (7)
C7	0.0326 (9)	0.0426 (11)	0.0382 (10)	−0.0054 (8)	−0.0048 (8)	−0.0017 (8)
C8	0.0295 (8)	0.0349 (9)	0.0276 (8)	0.0026 (7)	−0.0017 (7)	0.0004 (7)
C9	0.0279 (8)	0.0325 (9)	0.0276 (8)	0.0019 (7)	−0.0002 (6)	0.0012 (7)
C10	0.0415 (10)	0.0380 (10)	0.0361 (9)	−0.0029 (8)	−0.0033 (8)	0.0086 (8)
C11	0.0437 (11)	0.0387 (10)	0.0456 (11)	−0.0133 (8)	−0.0023 (9)	0.0073 (8)

### Bis(2,6-diamino-4-chloropyrimidin-1-ium) naphthalene-1,5-disulfonate (II) Geometric parameters (Å, º)

**Table:**

Cl1—C4	1.7290 (19)	S1—O3	1.4431 (16)
N1—C6	1.352 (2)	S1—O1	1.4550 (15)
N1—C2	1.363 (2)	S1—O2	1.4584 (15)
N1—H1	0.8600	S1—C8	1.7749 (17)
N2—C2	1.319 (3)	C7—C8	1.361 (3)
N2—H2A	0.8600	C7—C11^i^	1.403 (3)
N2—H2B	0.8600	C7—H7	0.9300
N3—C2	1.321 (3)	C8—C9	1.433 (2)
N3—C4	1.335 (3)	C9—C10	1.419 (3)
N6—C6	1.311 (2)	C9—C9^i^	1.427 (3)
N6—H6A	0.8600	C10—C11	1.361 (3)
N6—H6B	0.8600	C10—H10	0.9300
C4—C5	1.361 (3)	C11—C7^i^	1.403 (3)
C5—C6	1.402 (3)	C11—H11	0.9300
C5—H5	0.9300		
			
C6—N1—C2	121.84 (16)	O3—S1—O1	113.34 (10)
C6—N1—H1	119.1	O3—S1—O2	113.21 (10)
C2—N1—H1	119.1	O1—S1—O2	111.10 (9)
C2—N2—H2A	120.0	O3—S1—C8	105.66 (8)
C2—N2—H2B	120.0	O1—S1—C8	106.56 (8)
H2A—N2—H2B	120.0	O2—S1—C8	106.34 (9)
C2—N3—C4	115.40 (16)	C8—C7—C11^i^	120.15 (17)
C6—N6—H6A	120.0	C8—C7—H7	119.9
C6—N6—H6B	120.0	C11^i^—C7—H7	119.9
H6A—N6—H6B	120.0	C7—C8—C9	121.31 (16)
N2—C2—N3	121.08 (18)	C7—C8—S1	118.35 (13)
N2—C2—N1	116.65 (18)	C9—C8—S1	120.31 (13)
N3—C2—N1	122.27 (18)	C10—C9—C9^i^	119.00 (19)
N3—C4—C5	127.02 (18)	C10—C9—C8	123.19 (15)
N3—C4—Cl1	114.47 (14)	C9^i^—C9—C8	117.8 (2)
C5—C4—Cl1	118.51 (16)	C11—C10—C9	120.90 (17)
C4—C5—C6	115.80 (18)	C11—C10—H10	119.6
C4—C5—H5	122.1	C9—C10—H10	119.6
C6—C5—H5	122.1	C10—C11—C7^i^	120.83 (18)
N6—C6—N1	118.48 (17)	C10—C11—H11	119.6
N6—C6—C5	123.87 (18)	C7^i^—C11—H11	119.6
N1—C6—C5	117.64 (16)		
			
C4—N3—C2—N2	−179.1 (2)	O3—S1—C8—C7	−116.17 (16)
C4—N3—C2—N1	0.7 (3)	O1—S1—C8—C7	122.99 (16)
C6—N1—C2—N2	−179.4 (2)	O2—S1—C8—C7	4.41 (18)
C6—N1—C2—N3	0.8 (3)	O3—S1—C8—C9	61.75 (17)
C2—N3—C4—C5	−1.8 (3)	O1—S1—C8—C9	−59.08 (16)
C2—N3—C4—Cl1	178.00 (16)	O2—S1—C8—C9	−177.67 (14)
N3—C4—C5—C6	1.3 (3)	C7—C8—C9—C10	179.44 (18)
Cl1—C4—C5—C6	−178.48 (14)	S1—C8—C9—C10	1.6 (2)
C2—N1—C6—N6	178.92 (19)	C7—C8—C9—C9^i^	−0.6 (3)
C2—N1—C6—C5	−1.3 (3)	S1—C8—C9—C9^i^	−178.50 (17)
C4—C5—C6—N6	−179.95 (19)	C9^i^—C9—C10—C11	−0.3 (3)
C4—C5—C6—N1	0.3 (3)	C8—C9—C10—C11	179.62 (19)
C11^i^—C7—C8—C9	0.8 (3)	C9—C10—C11—C7^i^	0.1 (3)
C11^i^—C7—C8—S1	178.73 (16)		

Symmetry code: (i) −*x*+2, −*y*+1, −*z*+1.

### Bis(2,6-diamino-4-chloropyrimidin-1-ium) naphthalene-1,5-disulfonate (II) Hydrogen-bond geometry (Å, º)

**Table:**

*D*—H···*A*	*D*—H	H···*A*	*D*···*A*	*D*—H···*A*
N1—H1···O1	0.86	1.92	2.708 (2)	152
N2—H2*A*···O2^ii^	0.86	2.08	2.868 (3)	152
N2—H2*B*···O2	0.86	2.10	2.953 (2)	174
N6—H6*A*···N3^iii^	0.86	2.25	2.943 (2)	138
N6—H6*B*···O3^iv^	0.86	2.01	2.808 (2)	154

Symmetry codes: (ii) −*x*+1, −*y*+1, −*z*; (iii) *x*+1/2, −*y*+3/2, *z*+1/2; (iv) *x*−1/2, −*y*+3/2, *z*+1/2.
